# Supplementary material for: Near-field thermophotovoltaics for efficient heat to electricity conversion at high power density
Source: Nat Commun. 2021 Jul 16;12:4364. doi: 10.1038/s41467-021-24587-7 (PMC8285488; doi:10.1038/s41467-021-24587-7)
Supplement: Supplementary file 1 — Supplementary Information [file 41467_2021_24587_MOESM1_ESM.pdf]

Supplementary information for

Near-field thermophotovoltaics for efficient heat to electricity conversion at high power density

Rohith Mittapally[†,1], *Byungjun Lee*[†,2], *Linxiao Zhu*[3], *Amin Reihani*[1], *Ju Won Lim*[4], *Dejiu Fan*[2], *Stephen R. Forrest*[*,2,4,5], *Pramod Reddy*[*,1,2,4] *and Edgar Meyhofer*[*,1,6]

[1]Department of Mechanical Engineering, University of Michigan, Ann Arbor, MI 48109, USA

[2] Department of Electrical Engineering and Computer Science, University of Michigan, Ann Arbor, MI 48109, USA

[3]Department of Mechanical Engineering, The Pennsylvania State University, University Park, PA 16802, USA

[4]Department of Materials Science and Engineering, University of Michigan, Ann Arbor, MI 48109, USA

[5]Department of Physics, University of Michigan, Ann Arbor, MI 48109, USA

[6]Department of Biomedical Engineering, University of Michigan, Ann Arbor, MI 48109, USA

†These authors contributed equally to this paper.

*email: stevefor@umich.edu, pramodr@umich.edu, meyhofer@umich.edu

Supplementary Note 1. Fabrication of the suspended thermal emitter

A schematic diagram of the fabrication process for the emitter device is shown in Supplementary Fig. 1a. (Step 1) A commercially available 4” SOI wafer (Ultrasil LLC) with a heavily doped (P-type B-doped) device layer and a thickness of 60 ± 1 μm is chosen. The specified resistivity of the top device layer is <0.005 Ω.cm, which corresponds to a doping level ~3 × 10^{19} cm^{-3}. The handle layer is undoped with a thickness of 500 ± 10 μm. The oxide layer thickness between the device layer and the handle layer is 2 μm ± 5%, which acts as an etch stop for fabricating the suspended emitter. (Step 2) The top surface is patterned using standard lithographic techniques to define the mesa surface. This pattern is etched to a depth of 15 μm in a deep reactive ion etching (DRIE) tool. (Step 3) A three-layer metal contact pad (Ti/Pt/Au – 10/30/200 nm) is evaporated and lifted-off to form electrical contacts to the two legs of the emitter. The wafer is then lithographically patterned and etched (~45 μm deep) in a DRIE tool until the BOX layer is reached. This step defines the beam structures and isolates the two electrical contacts. (Step 4)

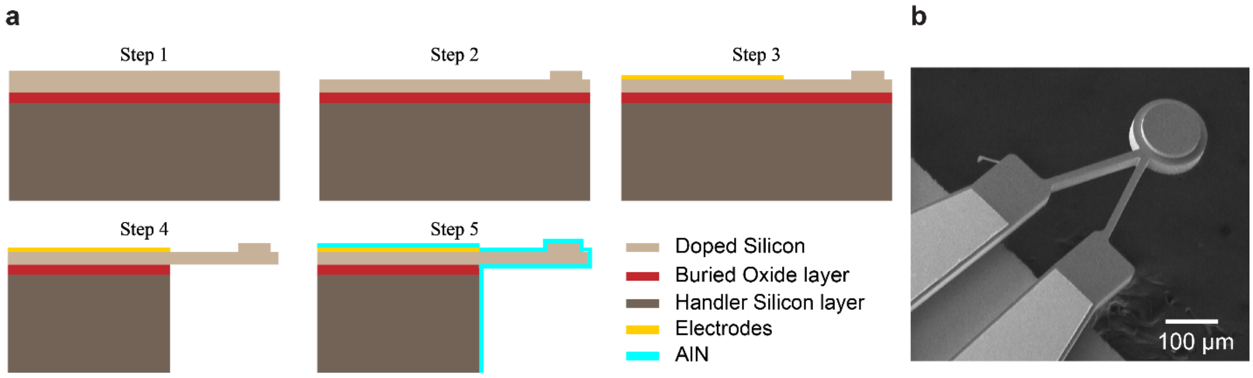

***Supplementary Figure 1. Fabrication process for the suspended silicon emitter. a**, Steps followed to suspend silicon emitter from two isolated beams. **b**, Scanning electron micrograph of the fabricated structure.*

Next, we proceed to release the emitter device to form a suspended structure. The front-side of the wafer is first coated with a thick photoresist (AZ-9260) for protection. The backside is subsequently coated with a thick photoresist (10 μm) and patterned to expose windows for etching. The exposed silicon undergoes a through-etch (DRIE – 500 μm deep) process until the stop BOX layer is reached. The residual BOX layer is then etched using a recipe that selectively etches silicon dioxide, resulting in a suspended monolithic silicon emitter. Next, the released chips are thoroughly cleaned in hot Piranha to remove any organic contaminants while retaining the smoothness of the mesa surface. (Step 5) A 10 nm-thick layer of aluminum nitride (AlN) is coated

using an atomic layer deposition (ALD) tool. In Supplementary Fig. 1b we show a SEM image of a fabricated silicon emitter device, suspended from the base through two silicon beams.

Supplementary Note 2. Fabrication of the InGaAs photovoltaic cell

A schematic diagram of the fabrication process for the PV cell is shown in Supplementary Fig. 2. (Step 1) The PV cell fabrication starts with the epitaxial growth of a lattice-matched, inverted *P-n-N* heterostructure on a 2 inch, 350 µm thick, single-side polished, Zn-doped, (100) InP substrate using a GENxplore Molecular Beam Epitaxy system (Veeco Corp.). The structure comprises of a 200 nm undoped InP buffer layer, 200 nm Be-doped (1×10^{18} cm^{-3}) $In_{0.53}Ga_{0.47}As$ (InGaAs) top contact layer, 200 nm Be-doped (1×10^{18} cm^{-3}) InP front window layer, 1 µm Si-doped (1×10^{17} cm^{-3}) InGaAs absorption layer, 100 nm Si-doped (1×10^{18} cm^{-3}) InP back window layer and 100 nm Si-doped (1×10^{18} cm^{-3}) InGaAs bottom contact layer. (Step 2) The PV structure is rinsed in buffered HF (BHF) for 90 s, followed by deposition of an Au/Parylene-C (400/1000 nm) bottom contact and bonding layer via sputtering and physical vapor deposition. Subsequently, the wafer is bonded, via thermal compression bonding (150˚ C, 500 kPa, 5 min) onto a 500 µm Si handle coated with 1 µm Parylene-C. (Step 3) The Si/Parylene/Au/PV/InP stack is submerged in HCl: H_2O (1:1) solution with Si handle facing down for 24 hrs, to remove InP substrate. After removal of the InP substrate, the top surface of the PV device was immediately cleaned using NH_4OH brushing and megasonic cleaning. (Step 4) The PV mesa is defined using standard photolithography and wet etching. Citric acid: H_2O_2 (4:1) and HCl: H2O (1:1) solutions are used for etching the InGaAs and InP layers, respectively. (Step 5) The Ti/Pt/Au (10/30/1000 nm) top contact layer is patterned via standard photolithography and electron beam deposition. (Step 6) A polyimide insulating layer (PI-2555) was spin-coated and annealed at 200°C for an hour, and then patterned via standard lithography and RIE plasma etching to define the device active area and bottom contact pad openings. (Step 7) The Ti/Au (5/1000 nm) top contact pad is patterned via standard photolithography and electron beam deposition. Finally, the top InGaAs contact layer is etched using a citric acid: H_2O_2 (4:1) solution to expose the active area of the PV cell and prevent parasitic absorption.

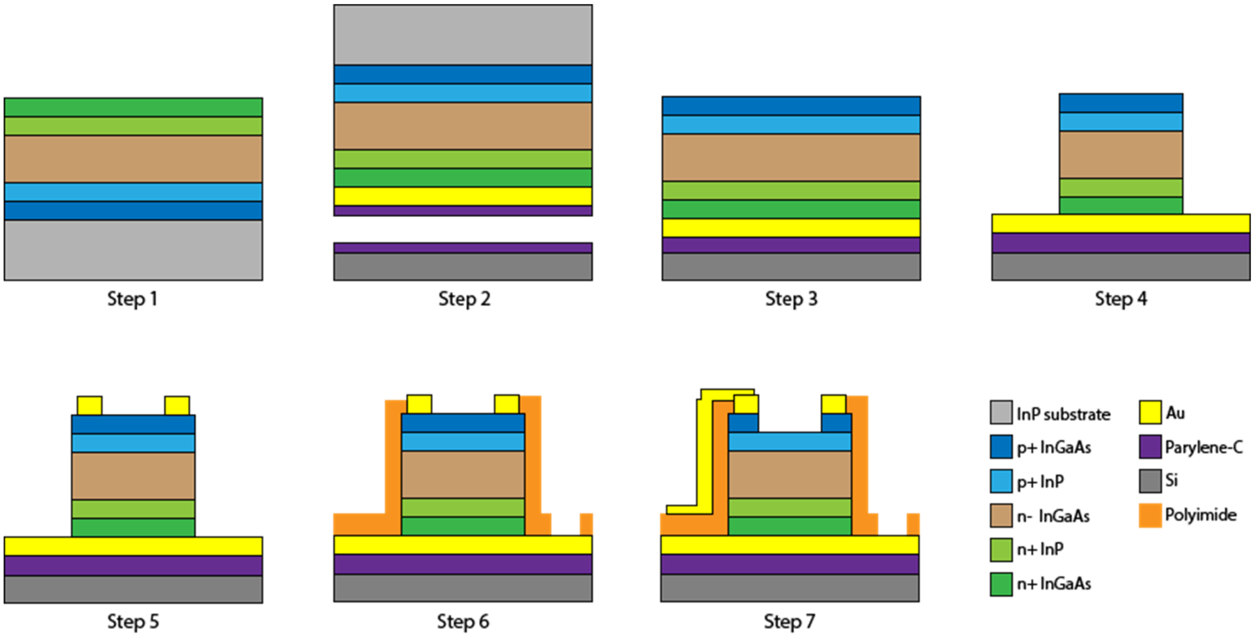

***Supplementary Figure 2*. *Fabrication process for the InGaAs PV cell*.** *The various steps involved in the fabrication of the PV cell are illustrated.*

Supplementary Note 3. Thermal and mechanical modelling of silicon emitter and quantification of heat loss through various pathways

Thermal characteristics

To understand the thermal and mechanical characteristics of the emitter at high temperatures, we performed FEM analysis, using COMSOL Multiphysics, on the structure shown in Supplementary Fig. 3a. Specifically, we employed the heat transfer, solid mechanics, electric currents and surface-to-surface radiation modules, which were coupled to the physics modules of thermal expansion and electromagnetic heating by using the relevant boundary conditions. In the heat transfer module, a constant temperature (300 K) condition was applied on surfaces A and B (see Supplementary Fig. 3a). Bipolar voltages of V+(~3.75) and V–(~-3.75 V) were also applied on surfaces A and B respectively, which were then used to solve for the resulting currents to determine the Joule heating in the electromagnetic heating module. To account for the radiative coupling to the environment, a nominal emissivity of 0.7 is applied on all the surfaces of the emitter, while the environmental temperature was set to 300 K. The thermal conductivity of silicon as a function of temperature was approximated from that of bulk intrinsic silicon as described in ref.[1]. Supplementary Fig. 3b shows the resulting temperature rise (color scale in kelvin) in the emitter structure, with the mesa being at the highest temperature of 1292 K. The distributed Joule heating

in the beams results in a temperature gradient that is nearly exclusively spread along the length of the beams, while the mesa surface is seen to be isothermal with less than 4 K temperature change along the two lines shown in Supplementary Fig. 3b. A transient thermal analysis was performed on the structure to estimate the time constant of the emitter. The temperature change with respect to time is shown in Supplementary Fig. 3c, indicating that the thermal time constant is ~20 ms. We note that all the near-field measurements were carried out after allowing for a thermal stabilization time of at least 3 s.

An important consideration in the design of thermal emitters is the possible distortion of the emitter due to thermal expansion and bi-material effects. We note that by suspending a monolithic silicon emitter, instead of creating a doubly clamped structure; we reduce potential bending and buckling effects. In Supplementary Fig. 3d, we show the computed absolute displacements for an emitter heated to 1292 K. Thermal expansion occurs predominantly in the direction along the length of the beams with a maximum displacement of ~1 μm at the distal end of the cantilever (Supplementary Fig. 3d) and the mesa surface is expected to remain flat at all temperatures. We note that, for simplifying the simulations, we did not consider the temperature-dependence of resistivity and emissivity of doped silicon in this simulation, but this simplification is not expected to affect any of our conclusions.

Finally, we note that the heat dissipated in the beams is lost through conduction along the beams and radiation from all the surfaces to the surroundings. The contribution of different pathways is affected by the temperature and the gap size. For example, at $T_{emitter}$ = 1270 K achieved by dissipating 550 mW in the emitter and a gap size of 100 nm, the total radiative heat from the surfaces facing the PV cell is 2 mW (0.4%) and from the backside and the side walls of the emitter is ~10 mW (1.8%). The major heat pathway is through conductance along the beams of 537 mW (98%).

Mechanical stiffness

To precisely maintain the gap size between large planar surfaces, it is important to design a stiff cantilever to minimize deflections at the smallest gap sizes and avoid snap-in (for example due to electrostatic attraction) at nanometer-scale gap sizes. Our emitter consists of a large circular island suspended by two beams, each 20 μm wide, 270 μm long and 45 μm deep. The resulting stiffness of the emitter as a function of temperature was also calculated using a FEM model similar to that

described in the previous section. The temperature-dependent elastic modulus (E) of silicon is estimated from the semi-empirical formula obtained from ref.[2].

$$E(T) = E_0 - BTe^{-T_0/T} .$$

Here, T is the temperature of silicon, E_0 = 167.5 GPa is the elastic modulus at 0 K, B = 15.8 MPa K^{-1} and T_0 = 317 K are the fitting parameters[2]. A constant force, F = 10 mN, was applied on mesa normal to the surface (Supplementary Fig. 3a), while the emitter is heated, using the model described in the previous section, by applying a voltage V that is varied from 1.5 V to 3.9 V in steps of 0.1 V. The resulting displacement (δ) at point 'A' (Supplementary Fig. 3e) is monitored at these temperatures and the stiffness is calculated as $k = F/\delta$ (Supplementary Fig. 3f). This was found to be ~2.15 kN/m at 800 K and reduced by ~5% as the temperature is increased to 1300 K.

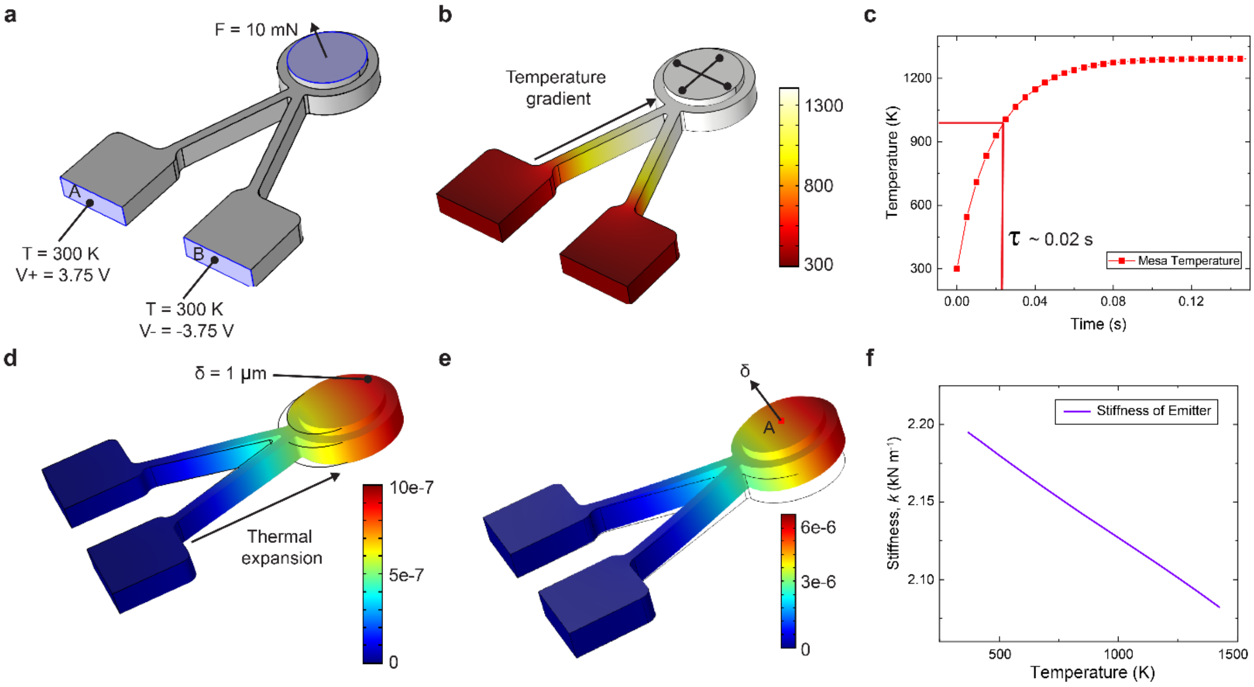

***Supplementary Figure 3. Thermal and mechanical characteristics of the emitter. a**, Boundary conditions applied for the FEA analysis. **b**, Temperature distribution on the emitter when heated by joule heating to 1292 K (scale bar in K). **c**, Temporal response of the emitter showing a thermal time constant of 20 ms. **d**, Thermal expansion of the emitter (color bar in m). **e**, The displacement, at point 'A' on the surface, resulting from a force F = 10 mN, applied on mesa surface as shown in Supplementary Fig. 3a. **f**, Stiffness of the emitter as a function of temperature.*

Supplementary Note 4. Electro-thermal characteristics of emitter

In this study, controlled heating of the emitter is achieved by employing heavily-doped silicon (~3 × 10^{19} cm^{-3}) as the resistive material. The electrical circuit used in this study involves applying a

bipolar voltage using a DC power supply and measuring the electrical current through the circuit using a multimeter, as shown in the inset of Supplementary Fig. 4a. In Supplementary Fig. 4a, we plot the resistance of the emitter as a function of the power dissipated, while the corresponding temperature of the emitter is plotted on the secondary axis (the details of this temperature measurement are provided in supplementary section 6). An initial increase in the resistance of the doped silicon is observed when the emitter is heated, with the resistance reaching a maximum at ~1200 K, beyond which it starts to decline. This trend can be understood by considering the temperature-dependent electrical resistivity ($\rho(T)$) of doped silicon as $\frac{1}{n(T)e\mu(T)}$, where $n(T)$ is the temperature-dependent number of charge carriers, e is the elementary charge and $\mu(T)$ is the temperature-dependent charge carrier mobility. At relatively low temperatures, $\rho(T)$ is dominated by the temperature dependency of carrier mobility ($\mu(T)$) which decreases with increasing temperature[3]. As the temperature is increased, the number of thermally generated intrinsic carriers ($n(T)$) increases significantly[4], causing $\rho(T)$ to drop with temperature. Such a behavior, called thermal runaway, is observed in doped silicon resistors as described in ref.[4]. An additional observation is that the resistance curve displays hysteresis after few hours of operation at high temperatures, and thus, it is not feasible to use the resistance as an indicator of temperature, instead, we measure the temperature in an independent experiment (supplementary note 6).

To reduce the electrostatic forces between the parallel plates of the emitter and PV cell during the near-field approach, we apply a bipolar voltage across the device bringing the mesa potential close to ground. To test the effectiveness of this approach, we fabricated a few 3-terminal devices and tested them in representative conditions. In Supplementary Fig. 4b two examples are shown, one by applying a bipolar voltage (case a) and the other by passing current (case b) from a Keithley 6220 current source (insets of Supplementary Fig. 4a) through the two outer terminals of the device, while monitoring the voltage (V_{Mon}) on the mesa with the third terminal in the center. It can be seen that for case a, V_{Mon} remains close to zero potential while a continuous increase is seen for case b, indicating that the higher electrostatic force for case b could lead to snap-in of the devices at higher gap sizes than that for case a.

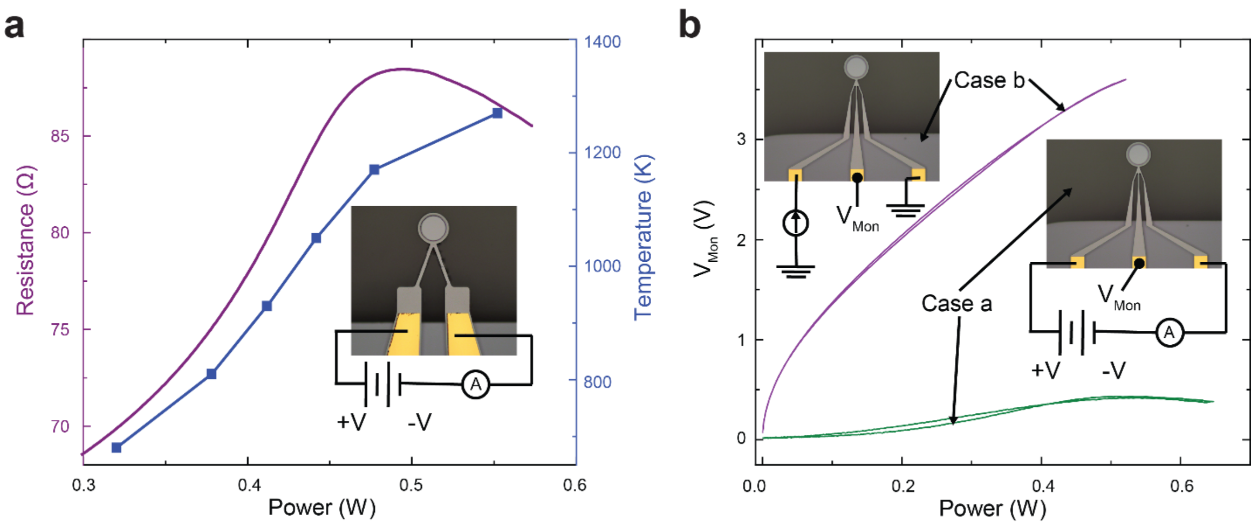

***Supplementary Figure 4. Electro-thermal characteristics of the emitter. a**, Resistance (violet solid line, left axis) and experimentally measured temperature (blue squares, data points connected by a straight line, right axis), as a function of the power dissipated in the emitter. Inset shows the electrical connections **b**, Measured voltage of the mesa (V_{Mon}) as a function of power dissipation in a representative 3-terminal device for two cases (bipolar and unipolar heating).*

Supplementary Note 5. Thermal modelling to determine temperature rise of the PV cell

In our experiments, the PV cell is mounted on the bottom assembly of our custom-built nanopositioner as shown in Supplementary Fig. 5a. As can be seen, the PV cell substrate (10 × 8 × 0.5 mm, red square) is mounted on a DIP chip carrier (shown in yellow, 'B') with silver paste which in-turn is attached to an aluminum platform. From a simple one-dimensional thermal model, we estimate the thermal conductances from point 'A' to point 'F' (a large heat-sink at ambient temperature). The smallest thermal conductance is between point 'A' to point 'B'. In other words, the thermal resistance is dominated by the structure shown in Supplementary Fig. 5b. In order to identify the temperature rise of the PV cell due to the heat transfer from the emitter, we then performed FEM analysis, solving the thermal conduction equation for a representative PV cell when it is illuminated by the hot emitter. Supplementary Fig. 5b shows the simulated structure consisting of a thick silicon substrate (0.4 × 0.4 × 0.2 mm). Thin layers of parylene (2 µm), gold (400 nm), GaAs (1.5 µm) and gold (1 µm) are included to represent a realistic model of the PV cell consistent with the description in supplementary note 2 above. The bottom surface is maintained at a constant temperature of 300 K, while heat (Q_{input}) is applied on the top surface of the PV cell at its center. The corresponding temperature rise (ΔT_{PV}) is calculated for different inputs of heat (Supplementary Fig. 5c). The estimated radiative heat transfer (Q_{RHT}) to the PV cell at the highest temperature (1270 K) achieved in this work and a gap of 100 nm, is ~2 mW. Thus,

based on this analysis the temperature rises of the PV cell in the experiments performed in this study are expected to be less than 1 K. We note that in the real experiments, the PV cell may be heated to a slightly higher temperature (~5 K), possibly due to heat emission from the beams of the emitter which is not considered in the calculation of Q_{RHT} and interfacial thermal resistances in the multilayers of the PV cell that are also not included in the simulation.

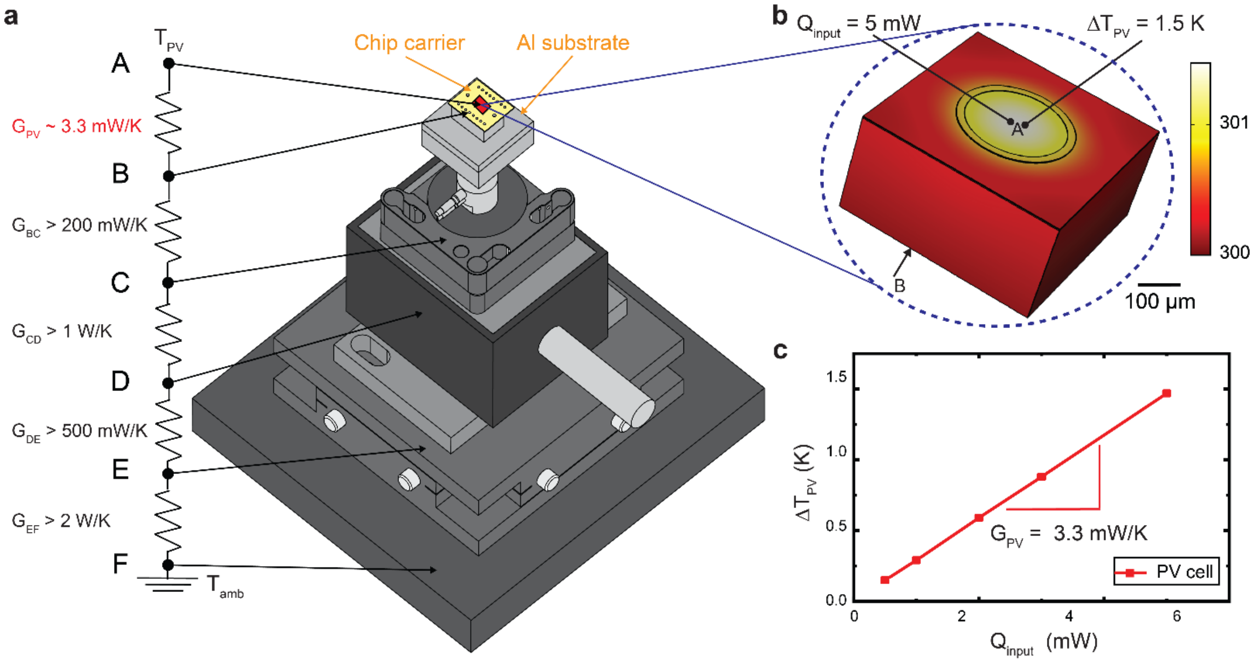

Supplementary Figure 5. Thermal management of PV cell. a*, Schematic of the bottom assembly of the nanopositioner onto which the PV cell is mounted. Typical thermal conductances along different sections of this assembly are shown.* ***b****, Boundary conditions applied for FEA of the temperature distribution of the PV cell due to heat transfer from the mesa of the emitter to the PV cell.* ***c****, Temperature increase at point 'A' (Supplementary Fig. 5a) at the center of the top surface of the PV cell, as a function of heat input to the PV cell.*

Supplementary Note 6. Emitter Temperature Measurement via Scanning Thermal Microscopy

The temperature of the emitter as a function of dissipated power was measured on the same device after the near-field experiments were performed, using a quantitative high-temperature scanning thermal microscopy (SThM) technique in an ultra-high vacuum environment following an approach described in ref.[5]. Briefly, a custom-fabricated SThM probe with an integrated Pt serpentine resistor that serves both as a heater and thermometer was used for these measurements. The temperature at the probe's tip (T_{tip}) can be accurately quantified with the Pt thermometer.

Further, the same Pt resistor can be used to simultaneously heat the probe tip by inputting heat ($\dot{q}_{tip}$) into the tip. In our measurements the probe was placed close to the center of the emitter's mesa but at a distance of ~5 µm above the surface, with the orientation shown in Supplementary Fig. 6a. Then the probe was moved towards the mesa to contact the emitter. The thermal behavior of the resulting structure can be represented by the DC equivalent thermal circuit shown in Supplementary Fig. 6b, where R_C and R_{TS} are the cantilever thermal resistance and tip-sample thermal contact resistance, respectively. Given the value of R_{TS}, the surface temperature (T_s) can be calculated based on the measurement of T_{tip} using

$$T_S = \frac{R_{TS}}{R_C}\left(T_{tip-contact} - T_{tip-retract}\right) + T_{tip-contact}$$

where $T_{tip\text{-}retract}$ and $T_{tip\text{-}contact}$ refer to SThM tip temperatures when retracted (out of contact) and when in contact with the mesa. We note that upon contact of the probe and emitter, an additional heat transfer path is provided by which the emitter's temperature is reduced. However, this decrease is less than 0.4% of the total temperature rise of the emitter due to the significantly larger thermal resistance of the additional path (R_{TS} + R_C) compared to the thermal resistance of the emitter. Hence this effect can be neglected.

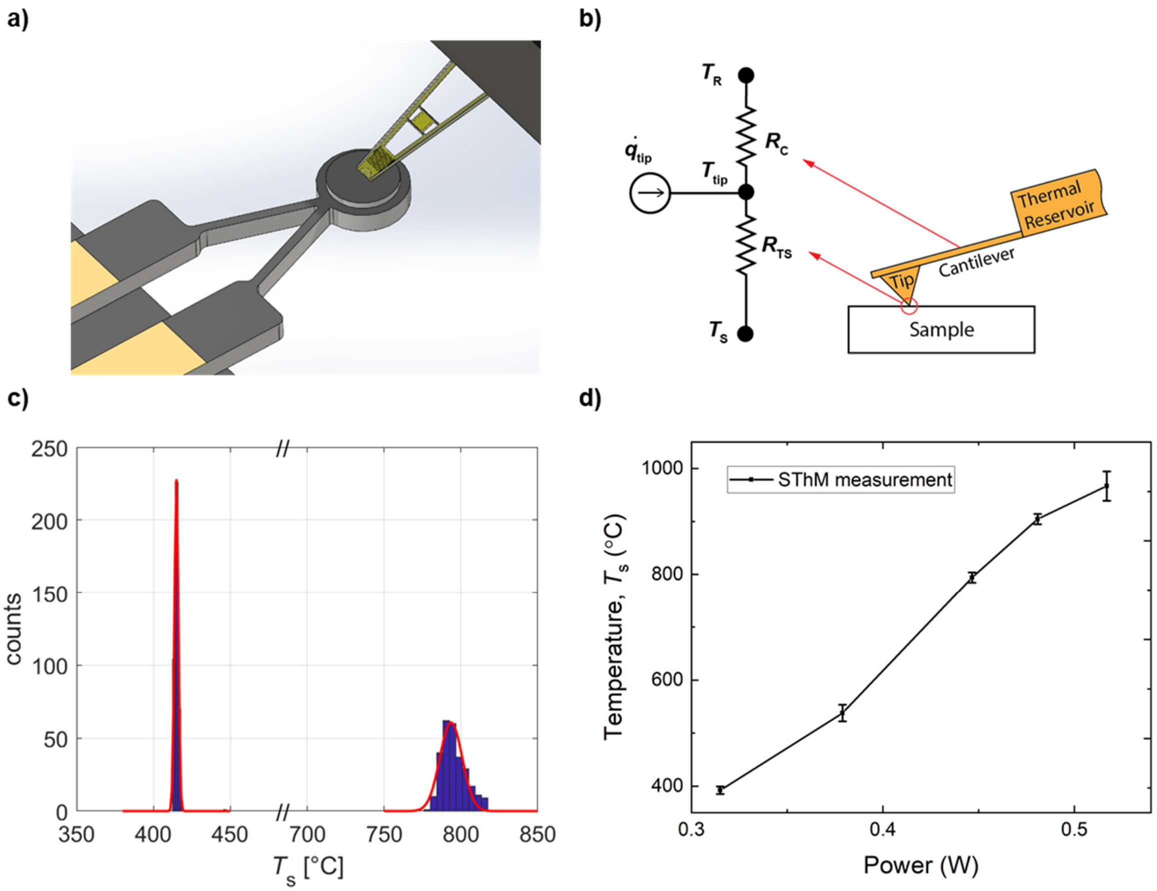

Supplementary Figure 6. Temperature measurement of emitter using SThM. (a) *Schematic of the SThM measurement orientation on the emitter mesa.* ***(b)*** *DC-equivalent thermal resistance network of the SThM probe in contact with a sample, and* ***(c)*** *histograms of surface temperature measurement using SThM at two different emitter powers of 0.314 W and 0.45 W showing mean temperature values of 414.8 °C and 794.1 °C and the standard deviation in the data is 1.6 K and 9.7 K, respectively.* ***(d)*** *Mean value of the emitter surface temperature as a function of dissipated power, error bars correspond to ±1σ (standard deviation).*

The contact resistance (R_{TS}) depends on the topography and local mechanical and thermal properties of the tip and contact point, therefore it typically varies from one measurement to another and can also change over time due to the mechanical drift of the SThM probe. For quantification of R_{TS}, we employed a DC + AC temperature measurement scheme (see ref.[5] for details) by applying a modulated heating power to the tip heater ($\dot{q}_{tip}$) at a frequency f_h = 4 Hz, and measuring both the DC tip temperature, and AC tip temperature amplitude at f_h. The change in the AC temperature amplitude when the probe is in contact compared to out of contact provides direct information about the value of R_{TS} based on a single-state dynamic thermal model as described in ref.[5]. Supplementary Fig. 6c shows the histogram of two measurements for an emitter

that dissipated powers of 0.314 W and 0.45 W, showing mean temperatures of 414.8 °C and 794.1 °C respectively. As can be seen, the measurement uncertainty increases at higher temperatures, because it is more difficult to achieve a stable contact at elevated temperatures. Finally, the mean value of emitter surface temperature as a function of emitter power is shown in Supplementary Fig. 6d. Since the temperature of the emitter as a function of dissipated power depends primarily on the temperature-dependent thermal conductivity of silicon, this relationship is expected to hold in our NF-TPV and the SThM temperature measurements. The non-monotonic increase in temperature with power may be attributed to increasing radiative coupling to the environment at higher temperatures and due to the temperature and position-dependent changes in material properties such as resistivity along the beams.

Supplementary Note 7. Modelling of the NF-TPV system

As described in the methods section of the main manuscript, our theoretical modelling involves calculating the radiative energy transfer between the emitter and the PV cell, and further estimating P_{MPP} from a PV cell model. We begin by describing our approach to modelling the spectral energy transfer.

Modelling Radiative Energy Transfer

The geometry and composition of the emitter and the PV cell used for modelling the radiative energy transfer are shown in Supplementary Fig. 7a. First, we model the permittivity of n-InGaAs by combining the intrinsic optical response of pure InGaAs, and free carrier absorption due to doping as:

$$\epsilon_{n-InGaAs}(\omega) = \epsilon_{InGaAs}(\omega), \text{ when } \omega \geq \omega_g$$

$$\epsilon_{n-InGaAs}(\omega) = \epsilon_{InGaAs}(\omega) + \epsilon_{Drude}(\omega), \text{ when } \omega < \omega_g,$$

where ω_g is the angular frequency corresponding to the bandgap of InGaAs.

For $\epsilon_{InGaAs}(\omega)$, at wavelengths shorter than 12 μm, we use tabulated values[6]. At wavelengths longer than 12 μm, the intrinsic optical response is due to phonons. We model $\epsilon_{InGaAs}(\omega)$ at these long wavelengths by interpolating between the permittivity of InAs[7] and the permittivity of GaAs[7] using the composition ($In_{0.53}Ga_{0.47}As$):

$$\epsilon_{InGaAs} = 0.53 \times \epsilon_{InAs} + 0.47 \times \epsilon_{GaAs}, \text{ when } \lambda > 12\ \mu m.$$

We use the following Drude term to account for the free-carrier absorption in the n-doped material:

$$\epsilon_{Drude} = -\frac{N_e e^2}{\epsilon_0 m_e^*} \frac{1}{\omega^2 - i\omega\Gamma_e},$$

where the electron concentration is given by $N_e = \frac{1}{2}\left(N_D + \sqrt{N_D^2 + 4n_i^2 e^{qV_J/k_B T_C}}\right)$, N_D is the donor concentration, and $n_i = 6.3 \times 10^{11}\ cm^{-3}$ is the intrinsic carrier concentration for InGaAs at room temperature[8]. Here, V_J is the voltage bias on the photovoltaic cell junction, and T_c is the temperature of the photovoltaic cell. The electron effective mass, m_e^*, is determined by using the dependence of electron effective mass on electron concentration[9]. The electron scattering rate is $\Gamma_e = \frac{e}{m_e^* \mu_e}$, where the electron mobility μ_e is obtained from ref.[10]. For a n-doping level $N_D = 1 \times 10^{17}\ cm^{-3}$, the effective mass is $m_e^* = 0.041\ m_e$, where m_e is the mass of electron, and the electron mobility is $\mu_e = 8727\ \frac{cm^2}{V \cdot s}$; At an n-doping of $1 \times 10^{18}\ cm^{-3}$, the effective mass $m_e^* = 0.0483\ m_e$, and electron mobility is $\mu_e = 5639\ \frac{cm^2}{V \cdot s}$.

To model the permittivity of doped InP, we use a similar approach by accounting for both the intrinsic optical response and free carrier effects. The permittivity of doped InP is:

$$\epsilon_{doped\ InP}(\omega) = \epsilon_{InP}(\omega), \text{ when } \omega \geq \omega_g$$

$$\epsilon_{doped\ InP}(\omega) = \epsilon_{InP}(\omega) + \epsilon_{Drude}(\omega), \text{ when } \omega < \omega_g\ ,$$

where ω_g is the angular frequency corresponding to the bandgap of InP. The permittivity for intrinsic InP is taken from ref.[7]. We use a Drude term to account for the free carrier optical response in doped InP:

$$\epsilon_{Drude} = -\frac{N_e e^2}{\epsilon_0 m_e^*} \frac{1}{\omega^2 - i\omega\Gamma_e} - \frac{N_h e^2}{\epsilon_0 m_h^*} \frac{1}{\omega^2 - i\omega\Gamma_h}.$$

Here, the electron and hole concentrations are given by

$$N_e = \frac{1}{2}\left(N_D - N_A + \sqrt{(N_D - N_A)^2 + 4n_i^2}\right) \text{ and}$$

$$N_h = \frac{1}{2}\left(N_A - N_D + \sqrt{(N_A - N_D)^2 + 4n_i^2}\right),$$

where N_D and N_A are donor and acceptor concentrations, respectively, and the intrinsic carrier concentration for InP[8] at room temperature is $n_i = 1.3 \times 10^7\ cm^{-3}$. The electron effective mass m_e^* is determined by using the dependence of electron effective mass on electron concentration[11]. The electron scattering rate is $\Gamma_e = \frac{e}{m_e^* \mu_e}$, where the electron mobility μ_e is obtained from ref.[12]. For the hole effective mass (ref. 9) we use $m_h^* = 0.6\ m_e$. The hole scattering rate is $\Gamma_h = \frac{e}{m_h^* \mu_h}$, where the hole mobility $\mu_h = 150 \frac{cm^2}{V \cdot s} \times \frac{1}{1+\left(\frac{N_A}{2\times 10^{17} cm^{-3}}\right)^{1/2}}$ and was obtained from ref.[13].

Based on the above parameters, we adopt the following values for our model: For the n-doped InP layer, with $N_D = 1 \times 10^{18}\ cm^{-3}$ and $N_A = 0$, the effective electron mass is $m_e^* = 0.0831\ m_e$, the electron mobility is $\mu_e = 2096 \frac{cm^2}{V \cdot s}$, the effective hole mass is $m_h^* = 0.6\ m_e$, and the effective hole mobility is $\mu_h = 150 \frac{cm^2}{V \cdot s}$. For the p-doped InP layer, with $N_A = 1 \times 10^{18}\ cm^{-3}$ and $N_D = 0$, the effective electron mass is $m_e^* = 0.08\ m_e$, electron mobility is $\mu_e = 5400 \frac{cm^2}{V \cdot s}$, the effective hole mass is $m_h^* = 0.6\ m_e$, and the effective hole mobility is $\mu_h = 46.4 \frac{cm^2}{V \cdot s}$. Finally, the permittivity for gold is obtained from ref[7].

Next, we describe the modelling of the emitter, where the circular mesa consists of a 60 μm thick layer of p-doped Si (doping concentration 3×10^{19} cm^{-3}), covered by 10 nm-thick layer of AlN on both sides. To account for the high-temperature operation of the emitter, a temperature-dependent permittivity is used to model the doped Si[14,15]. The optical properties of the aluminum nitride layer are modelled using tabulated permittivity at room temperature[16].

Using a scattering matrix formalism based on fluctuational electrodynamics, we then calculate the radiative energy transfer between any two layers in the multi-layer structure. In specific, to estimate P_{MPP}, we calculate the transmission probability from the mesa of the emitter to the active layer (junction) for both transverse-electric (TE) and transverse-magnetic (TM) modes, i.e., $\tau_{TE,mesa\to J}(\omega, k)$ and $\tau_{TM,mesa\to J}(\omega, k)$, where k denotes the parallel wave-vector. For simplicity, we use the following dimensionless transfer function.

$$\phi_{mesa\to J} = \left(\frac{\lambda}{2\pi}\right)^2 \int_0^\infty kdk\left[\tau_{TE,mesa\to J}(\omega,k) + \tau_{TM,mesa\to J}(\omega,k)\right].$$

Similarly, we denote the transfer function from the active layer of the photovoltaic cell to all the other layers of the photovoltaic cell as $\phi_{J\to O}$.

Next, to estimate the total energy transfer, Q_{RHT}, we calculate the transfer function from the emitter to the whole PV cell denoted as $\phi_{mesa\to C}$, where C denotes the whole PV cell. We denote the area of mesa as A_{mesa}. This calculation is performed in wavelengths ranging from 100 μm to 0.7 μm. Further, to calculate the contributions from the recessed 'rec' region of the emitter, we model it as multi-layer structure as shown in Supplementary Fig. 7b, similar to that followed for the mesa region. The corresponding transfer functions between the rec and active layer, rec and the whole PV cell are denoted by $\phi_{rec\to J}$ and $\phi_{rec\to C}$, respectively.

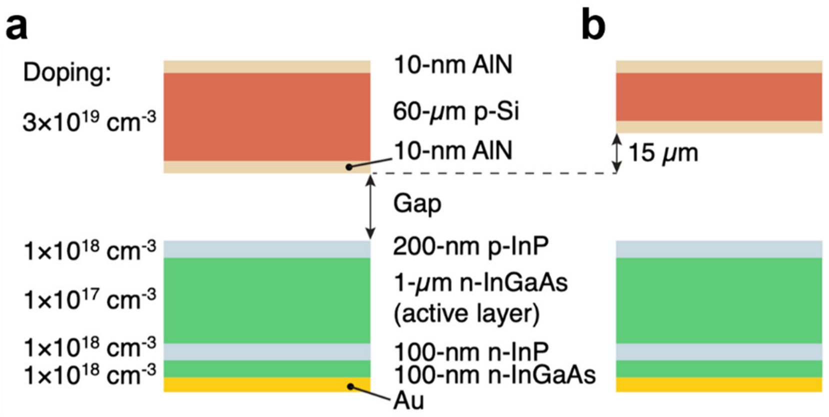

Supplementary Figure 7. Geometry for the PV cell and radiative energy transfer. ***a****, Schematic describing the geometry for the mesa of the emitter and the PV cell, including composition, doping concentration, and thickness of each layer.* ***b****, Schematic geometry for the recessed region of the emitter and PV cell.*

PV cell I-V response modelling

The *I-V* response of our PV cell is modelled using an equivalent circuit as shown in Supplementary Fig. 8, where the polarity for the current is defined such that both V and I are positive when the cell generates electricity. We use an experimentally obtained dark *I-V* response, which is measured under no illumination in vacuum at room temperature, to estimate the parasitic series resistance as $R_{series} = 85\,\Omega$ based on the slope of *I-V* curve at large forward voltage and a parasitic shunt resistance as $R_{shunt} = 5\times10^6\ \Omega$.

To calculate the total current generated in our PV cell, we include the current contributions from both the radiative and non-radiative recombination processes. First, we describe the current generated by radiative processes (I_{rad}) as:

$$I_{rad}(V_d) = e\int_{\omega_g}^{\infty} d\omega \frac{1}{\lambda^2}\Bigg[\left(VF_{mesa,J}\, A_{mesa}\phi_{mesa\to J} + VF_{rec,J}\, A_{rec}\phi_{rec\to J}\right)\left(\frac{1}{e^{\frac{\hbar\omega}{k_BT_E}}-1} - e^{-\frac{\hbar\omega - qV_J}{k_BT_C}}\right) + \left(VF_{mesa,J}\, A_{mesa}\phi_{O\to J}^{mesa} + VF_{rec,J}\, A_{rec}\phi_{O\to J}^{rec}\right)\left(e^{-\frac{\hbar\omega}{k_BT_C}} - e^{-\frac{\hbar\omega - qV_J}{k_BT_C}}\right)\Bigg]$$

where e is the elementary charge, T_C is the temperature of the cell including the junction, T_E is the temperature of the emitter, and subscript O denotes all the layers in the PV cell other than the active layer. Here, $VF_{\text{mesa,J}}$ and $VF_{\text{rec,J}}$ are the view factors associated with both the surfaces, the details of which are discussed in the next section. We assume the internal quantum efficiency of the PV cell to be 100%, or every photon absorbed in the active layer generates an e-h pair. We note that we have used the reciprocal relation for transfer functions, i.e. $\phi_{1\to 2} = \phi_{2\to 1}$.

Next, to account for current from non-radiative processes, we consider Auger recombination and Shockley-Read-Hall (SRH) recombination. The net recombination current associated with Auger recombination (I_{Auger}) is given by:

$$I_{Auger}(V) = eA_J\left(C_n n + C_p p\right)(np - n_i^2)t_J,$$

where C_n and C_p are the Auger recombination coefficients for electron and hole, respectively, n_i is the intrinsic carrier concentration at zero bias, and t_J and A_J are the thickness and area of the active layer of the photovoltaic cell, respectively. The combined Auger recombination coefficient is $C_n + C_p = 7 \times 10^{-29}\ cm^6/s$ as obtained from ref.[8]. We treat $C_n = C_p = 3.5 \times 10^{-29}\ cm^6/s$. For the active layer with n-doping,

$$n(V_J) = \frac{1}{2}\left(N_D + \sqrt{N_D^2 + 4n_i^2 e^{\frac{qV_J}{k_BT_C}}}\right), \text{ and } p(V_J) = \frac{1}{2}\left(-N_D + \sqrt{N_D^2 + 4n_i^2 e^{\frac{qV_J}{k_BT_C}}}\right).$$

The net recombination current associated with SRH process (I_{SRH}) is given by:

$$I_{SRH}(V_C) = eA_J S\frac{2(np - n_i^2)}{n + p + 2n_i},$$

where S is the effective surface recombination velocity. The total current (I_J) through the PV cell junction is then given by the sum of all the currents as:

$$I_J(V_J) = I_{rad}(V_J) - I_{Auger}(V_J) - I_{SRH}(V_J).$$

Further, by accounting for the series resistance and shunt resistance, the total current through the device and the total voltage across the device are:

$$I(V_J) = I_{rad}(V_J) - I_{Auger}(V_J) - I_{SRH}(V_J) - \frac{V_J}{R_{shunt}} \quad \text{and}$$

$$V = V_J - I(V_J) \cdot R_{series}.$$

The above two characteristic equations are used to obtain the *I-V* response of the PV cell. The surface recombination velocity $S \approx 3.5 \times 10^5 \frac{cm}{s}$ is a fitting parameter adjusted to best match the *I-V* curve obtained from our model with that of the experimentally measured dark *I-V* curve.

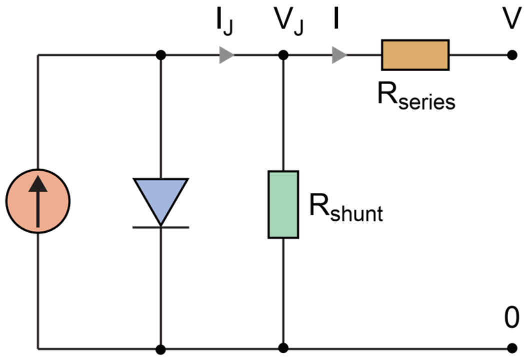

***Supplementary Figure 8. Equivalent circuit of the photovoltaic (PV) cell**. Schematic illustration of the equivalent circuit used to model the photovoltaic response of our cells.*

Supplementary Note 8. Effect of view factor in the near-field enhancement

In our theoretical model described in the previous section, we incorporate the effect of view factors between the surfaces on the emitter ('mesa' and the recessed region 'rec') and the surface of the PV cell (labeled 'J'). Specifically, we compute the view factors $VF_{mesa,J}$ and $VF_{rec,J}$, as a function of gap size. $VF_{mesa,J}$ is calculated by treating the geometry as two coaxial parallel discs[17] (mesa = 150 µm diameter and J = 190 µm diameter) as shown in the top inset of Supplementary Fig. 9a. Further, $VF_{rec,J}$ is calculated by approximating the geometry of recessed region and PV cell as two coaxial circular rings with inner diameter 150 µm and outer diameter 190 µm (bottom inset of

Supplementary Fig. 9a). An analytical expression for the view factor between circular rings is used as described in ref[18]. The variation of these two factors with gap size is plotted in Supplementary Fig. 9a. Since our calculation of radiative energy transfer between multilayer structures assumes the structures to be infinitely long in lateral directions, it is important to delineate the effect of view factors in the enhancement of power generation in the near-field. To this end, we plot the variation of theoretically computed P_{MPP} with the gap size. The data presented in Supplementary Fig. 9b (computed for T_{emitter} = 1270 K) illustrates that inclusion of the corrected view factor results in values of P_{MPP} that are somewhat different from those calculated with a view factor of 1 (infinite plates). In the far-field at 10 μm gap, ~10% variation in the power output could be seen, while the variation is less than 1% at smaller gaps. This is because the energy transfer in near-field is dominated by mesa whose view factor is close to 1 at these gaps as seen in Supplementary Fig. 9a. Thus, the effect of view factor on power enhancement at smaller gaps is negligible.

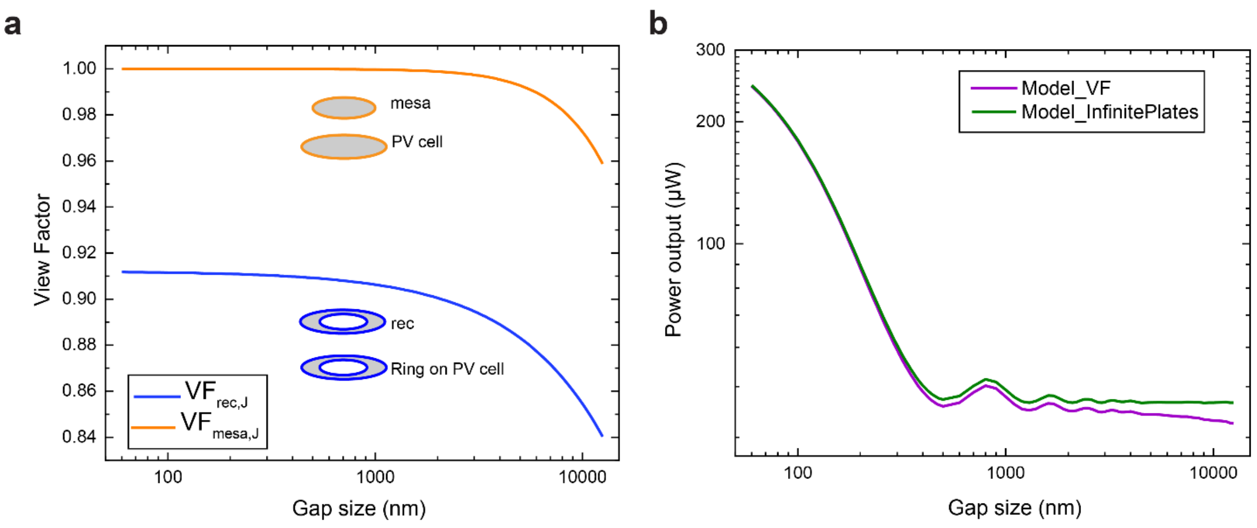

***Supplementary Figure 9. Effect of view factor on power enhancement**. **a,** View factors $VF_{\mathrm{mesa,J}}$ and $VF_{\mathrm{rec,J}}$ as a function of gap size between the emitter and PV cell. The orange curve corresponds to the view factor change as the gap between mesa and PV cell is reduced from above 10 μm to 60 nm, while the blue curve corresponds to the view factor change between the recessed region 'rec' (recessed by 15 μm) and a circular ring on PV cell with OD/ID of 190/150 μm. **b,** $P_{\mathrm{MPP,\,M}}$ as a function of gap size for two cases. Violet solid line corresponds to the incorporation of view factors calculated in Supplementary Fig. 9a, while the green curve corresponds to the assumption of view factor of 1 (infinite plates).*

Supplementary Note 9. Characteristics of the PV cell

The current-voltage response of the PV cell under dark conditions (referred to as Dark I-V) is shown in Supplementary Fig. 10a. For this measurement the PV cell was maintained at room temperature in a high vacuum of 1 μTorr. The voltage across the PV cell was varied from -0.5 V to 0.5 V in steps of 10 mV, while the current through the circuit was measured. The variation of fill-factor (*FF,* Supplementary Fig. 10b) with temperature is plotted along with the theoretical calculations. The small disagreement between measured and calculated *FF* may likely be due to the uncertainties in our modeling parameters such as the series and shunt resistances.

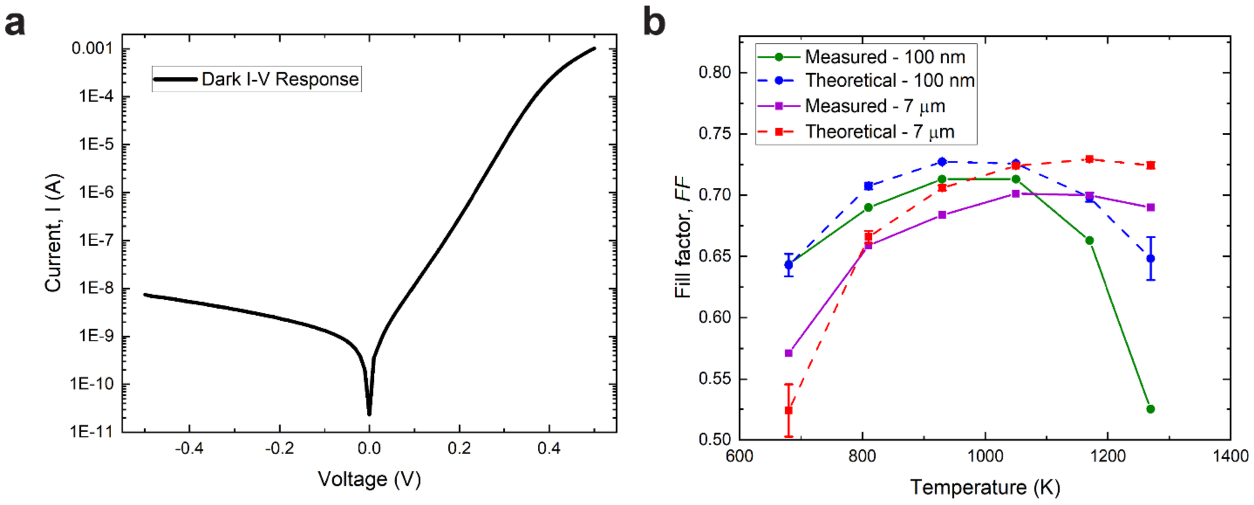

Supplementary Figure 10. Characteristics of the photovoltaic cell. a, *Dark I-V response of the PV cell.* ***b,*** *Measured and calculated fill factor (FF) as a function of temperature. Green circles and violet squares represent the experimental data points, while blue circles and red squares represent the calculated data points with the corresponding uncertainties, at gap sizes of 100 nm and 7 μm respectively. Solid and dashed lines added as a guide to the eye.*

Supplementary Note 10. Sub-band gap reflection from thin-film PV cell

The improvement in the efficiency of our NFTPV system was achieved by employing a thin-film PV cell as shown in Supplementary Fig. 11a. Typical bulk PV cells suffer from significant sub-band gap (SBG) absorption as a result of free-carrier absorption in heavily doped layers. The SBG suppression in the thin-film PV cell can be understood by comparing two structures: The current NFTPV system with $T_{emitter}$ = 1270 K at a gap size of 100 nm (Supplementary Fig. 11a) and a thick NFTPV structure, where the n-InP layer was assumed to have a thickness of 200 μm with $T_{emitter}$ = 1270 K at a gap size of 100 nm (Supplementary Fig. 11b). Using our theoretical model, we then

calculate the spectral energy transfers for both cases as shown in Supplementary Fig. 11c as a function of photon energy. The above-band gap flux is similar in both structures because the active layers are of identical thickness. A significant suppression of SBG is seen in a thin-film PV cell as compared to a bulk PV cell, because lower energy photons get absorbed in the 200 µm-thick n-InP layer before getting reflected by the gold layer. Thus, for the thin-film case, low energy photons get reflected back to the emitter improving the overall efficiency. The residual SBG absorption in the thin-film case is due to absorption in the gold layer (imperfect reflector).

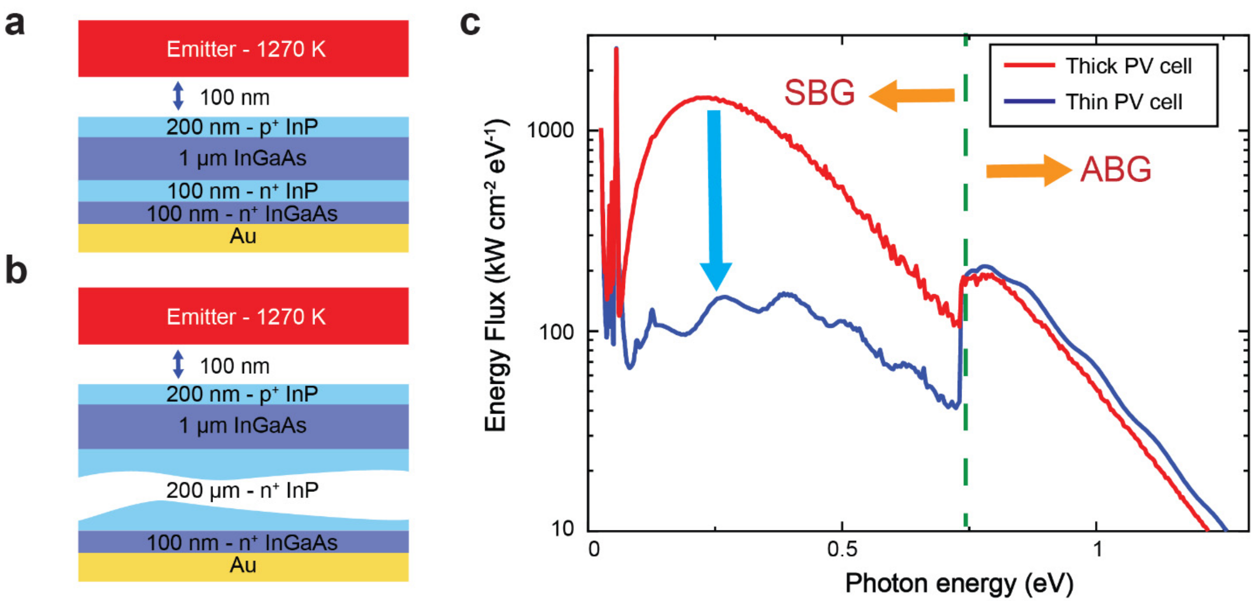

Supplementary Figure 11. Thin-film sub-band gap reflection a, *Schematic of the thin-film TPV system employed in this study showing the thicknesses of all the layers.* ***b,*** *Schematic of a hypothetical thick TPV structure, where the n-InP layer thickness is increased from 100 nm to 200 µm.* ***c,*** *Spectral energy transfer as a function of photon energy for a thin-film PV cell (solid blue line) and a bulk PV cell (solid red line).*

Supplementary References

1 Glassbrenner, C. J. & Slack, G. A. Thermal conductivity of silicon + germanium from 3 degrees K to melting point. *Phys. Rev.* **134**, 1058-1069 (1964).

2 Gysin, U. *et al.* Temperature dependence of the force sensitivity of silicon cantilevers. *Phys. Rev. B* **69** (2004).

3 Chapman, P. W., Tufte, O. N., Zook, J. D. & Long, D. Electrical properties of heavily doped silicon. *J. Appl. Phys.* **34**, 3291 (1963).

4 Chui, B. W. *et al.* Intrinsic-carrier thermal runaway in silicon microcantilevers. *Microscale Therm. Eng.* **3**, 217-228 (1999).

5 Reihani, A. *et al.* Quantifying the temperature of heated microdevices using scanning thermal probes. *Appl. Phys. Lett.* **118**, 163102 (2021).

6 Adachi, S. Physical properties of III-V semiconductor compounds : InP, InAs, GaAs, GaP, InGaAs, and InGaAsP. (Wiley, New York, 1992).

7 Palik, E. D. Handbook of optical constants of solids. (Academic Press, San Diego, 1998).

8 I. P.-T. Institute, NSM archive - Physical properties of semiconductors, 1998; The Ioffe Physical-Technical Institute of the Russian Academy of Sciences, 1998. http://www.ioffe.ru/SVA/NSM/Semicond.html.

9 Madelung, O. Semiconductors : data handbook, Edn. 3rd. (Springer, Berlin, 2004).

10 Pearsall, T. P. GaInAsP alloy semiconductors. (Wiley, New York, 1982).

11 Bugajski, M. & Lewandowski, W. Concentration-dependent absorption and photoluminescence of n-type Inp. *J. Appl. Phys.* **57**, 521-530 (1985).

12 Anderson, D. A., Apsley, N., Davies, P. & Giles, P. L. Compensation in heavily doped n-type Inp and Gaas. *J. Appl. Phys.* **58**, 3059-3067 (1985).

13 Wiley, J. D. Semiconductors and semimetals, Vol. 10. (eds. R.K. Willardson & A.C. Beer) 91-174 (Elsevier, 1975).

14 Fu, C. J. & Zhang, Z. M. Nanoscale radiation heat transfer for silicon at different doping levels. *Int. J. Heat Mass Tran.* **49**, 1703-1718 (2006).

15 Basu, S., Lee, B. J. & Zhang, Z. M. Near-field radiation calculated with an improved dielectric function model for doped silicon. *J. Heat Trans-T Asme* **132** (2010).

16 Adachi, S. Optical constants of crystalline and amorphous semiconductors: numerical data and graphical information. (Springer US, 1999).

17 Incropera, F. P., DeWitt, D. P., Bergman, T. L. & Lavine, A. S. Fundamentals of heat and mass transfer. (2007).

18 Sinnott, R. K. Coulson & Richardson's Chemical Engineering (7th ed.), Volume 1b: Heat and Mass Transfer: Fundamentals and Applications. 3-264 (2018).
