# Supplementary material for: Near-field thermophotovoltaics for efficient heat to electricity conversion at high power density
Source: Nat Commun. 2021 Jul 16;12:4364. doi: 10.1038/s41467-021-24587-7 (PMC8285488; doi:10.1038/s41467-021-24587-7)
Supplement: Supplementary file 3 — Solar Cells Reporting Summary [file 41467_2021_24587_MOESM3_ESM.pdf]

nature research

Corresponding author(s): Stephen R. Forrest
Pramod Reddy
Edgar Meyhofer

Solar Cells Reporting Summary

Nature Research wishes to improve the reproducibility of the work that we publish. This form is intended for publication with all accepted papers reporting the characterization of photovoltaic devices and provides structure for consistency and transparency in reporting. Some list items might not apply to an individual manuscript, but all fields must be completed for clarity.

For further information on Nature Research policies, including our data availability policy, see Authors & Referees.

Experimental design

Please check: are the following details reported in the manuscript?

1. Dimensions

Item	Reported	Details
Area of the tested solar cells	☒ Yes ☐ No	Cell diameter is provided on page 4 and methods section on page 18
Method used to determine the device area	☐ Yes ☒ No	Area of the circular device was calculated from the diameter

2. Current-voltage characterization

Item	Reported	Details
Current density-voltage (J-V) plots in both forward and backward direction	☐ Yes ☒ No	The current-voltage (I-V) plots in the forward direction are provided in Figs. 2b and 3a, while the data in backward direction is not presented as it is not relevant to power generation
Voltage scan conditions *For instance: scan direction, speed, dwell times*	☐ Yes ☒ No	A typical I-V scan was performed using a linear staircase sweep capability of Keithley 2401 Sourcemeter. The voltage is varied in the forward direction from 0 to 0.3 V in steps of 0.02 V with a dwell time of 1 ms.
Test environment *For instance: characterization temperature, in air or in glove box*	☒ Yes ☐ No	Test characterization information can be found on pages 4 and 5 of the manuscript
Protocol for preconditioning of the device before its characterization	☐ Yes ☒ No	All devices underwent solvent cleaning (10 minutes in acetone, followed by 5 minutes in isopropyl alcohol) before mounting them in the test setup.
Stability of the J-V characteristic *Verified with time evolution of the maximum power point or with the photocurrent at maximum power point; see ref. 7 for details.*	☐ Yes ☒ No	Since our study is a proof of principle, we did not perform stability studies on our TPV system.

3. Hysteresis or any other unusual behaviour

Item	Reported	Details
Description of the unusual behaviour observed during the characterization	☐ Yes ☒ No	No hysteresis was observed
Related experimental data	☐ Yes ☒ No	No hysteresis was observed

4. Efficiency

Item	Reported	Details
External quantum efficiency (EQE) or incident photons to current efficiency (IPCE)	☐ Yes ☒ No	We did not use EQE to calculate the power conversion efficiency. The details of our efficiency estimates can be found on page 8 of the manuscript
A comparison between the integrated response under the standard reference spectrum and the response measure under the simulator	☐ Yes ☒ No	The cells were not measured under the standard reference spectrum
For tandem solar cells, the bias illumination and bias voltage used for each subcell	☐ Yes ☒ No	We did not use tandem cells

5. Calibration

Item	Reported	Details
Light source and reference cell or sensor used for the characterization	☒ Yes ☐ No	The details of our custom-built light source (emitter) can be found on pages 4 and 18.

Confirmation that the reference cell was calibrated and certified

☐ Yes
☒ No

The characterization of the source temperature can be found in the methods section and supplementary note 6

Calculation of spectral mismatch between the reference cell and the devices under test

☐ Yes
☒ No

No reference cell was used in this study.

6. Mask/aperture

Size of the mask/aperture used during testing

☐ Yes
☒ No

No mask/aperture was used for testing

Variation of the measured short-circuit current density with the mask/aperture area

☐ Yes
☒ No

No mask/aperture was used for testing

7. Performance certification

Identity of the independent certification laboratory that confirmed the photovoltaic performance

☐ Yes
☒ No

The TPV cell was not sent out for certification

A copy of any certificate(s)
Provide in Supplementary Information

☐ Yes
☒ No

The TPV cell was not sent out for certification

8. Statistics

Number of solar cells tested

☐ Yes
☒ No

NFTPV measurements from one cell have been presented in the manuscript, and the electrical power output of 2 additional cells has been tested at a range of source (emitter) temperatures.

Statistical analysis of the device performance

☐ Yes
☒ No

No statistical analysis is reported in this study.

9. Long-term stability analysis

Type of analysis, bias conditions and environmental conditions
For instance: illumination type, temperature, atmosphere humidity, encapsulation method, preconditioning temperature

☐ Yes
☒ No

Long-term studies were not performed, but InGaAs cells are known to have long lifetimes
